# 

**Published:** 1902-05-15

**Authors:** 


### CONTENTS.

Some of the Things which Make a Dentist Professional, *By N. S. Hoff, D.D.S.* 217

Recurrence of Caries Under Gold Fillings, *By H. A. Smith, D.D.S.* 229

The Real Attitude of the Dental Specialty toward Medicine, - - *By Frank W. Sage, D.D.S.* 236

First Molar Necessary to Dentists' Financial Success. *By C. M. Wright, D.D.S.* 247

Bleaching Teeth with Peroxides of Hydrogen and Sodium, - *By Joseph Head, M.D., D.D.S.* 248

Using a Matrix Retainer in Plastic Filling. *By R. G. Hutchinson* 252

Treatment of Pyorrhea Alveolaris, *By Robert Good, D.D.S.* 254

EDITORIAL, - - - - - - - - - - 257

OBITUARY, - - - - - - - - - - 259

CORRESPONDENCE, - - - - - - - - - 260

ANNOUNCEMENTS, - - - - - - - - 262

INDENTS, - - - - - - - - - - 267

